# Uncovering the gender health data gap

**DOI:** 10.1590/0102-311XEN065423

**Published:** 2023-08-11

**Authors:** Vanessa di Lego

**Affiliations:** 1 Vienna Institute of Demography, Austrian Academy of Sciences, Vienna, Austria.

The way data are collected and interpreted by gender is subject to fundamental biases ^1^. This generates a gender health data gap that impacts all branches and levels of health research, including disease prevention and diagnosis, medical treatment for cancer, cardiovascular and Alzheimer’s disease ^2^ ^,^ ^3^.

The gender health data gap is characterized by ^1^ ^,^ ^4^ ^,^ ^5^: (1) missing or incomplete evidence for diseases that disproportionately impact women because of lack of funding or inclusion of women in clinical trials (henceforth called “type 1 problem”); (2) existing evidence is interpreted in light of men’s symptoms as default or textbook (henceforth called “type 2 problem”). The two problems are interrelated and stem from the perspective that male disease etiology and progression is the standard threshold and that differences between women and men are restricted to reproductive health ^6^ ^,^ ^7^. Because these issues are influenced by societal gender norms, I call this gender health data gap, and not only sex data gap, as further detailed as follows.

The type 1 problem is more prevalent in clinical research, as for over half a century the default model organism is based on male rodents ^1^ ^,^ ^6^. The lack of a female-based model contributed to the incomplete understanding of the etiology, symptomatology and treatment of a series of diseases in women ^1^. For example, women experience twice as much adverse drug reactions as men for over 86 different medications approved by the United States’ Federal Drug Administration (FDA), including antidepressants, analgesics, cardiovascular and anti-seizure drugs ^8^ ^,^ ^9^. However, most drugs are approved based on clinical trials that are either conducted solely on men or only include women in the first or second trial steps ^10^. As a result of such missing data on women, they face higher levels of over medication, adverse reaction, susceptibility for drug-induced liver injury, and dosage inaccuracy ^11^. Likewise, despite important differences between women and men in cognitive deterioration and brain atrophy rates, gender is rarely considered in the design and analysis of clinical trials ^12^. Lastly, women’s diseases are underfunded when compared with the burden of disease they experience and the lethality of conditions ^13^, including ovarian and cervical cancers ^14^, Alzheimer’s ^15^, and cardiovascular disease ^16^.

The type 2 problem presents itself more prominently among healthcare providers, where evidence is used inconsistently or influenced by subjective judgements. Some scholars call this “healthcare gender bias”, where women’s symptoms are dismissed or neglected, leading to delays in diagnosis ^3^ ^,^ ^5^. For example, women are less likely to receive the same care recommended by guidelines defined by the European Society of Cardiology (ESC) and the Acute Cardiovascular Care Association (ACCA), such as procedures like timely reperfusion therapy in the case of ST-elevation myocardial infarction (STEMI) and coronary angiography in case of non-ST-elevation myocardial infarction (NSTEMI) ^17^ ^,^ ^18^ ^,^ ^19^. Women are also less likely to receive or be referred to cardiac rehabilitation or be prescribed a statin or an angiotensin-converting-enzyme inhibitors (ACE inhibitor), which increase their 30-day mortality risk after a heart attack ^17^ ^,^ ^20^. As a consequence, deaths among women with acute myocardial infarction could be significantly prevented if the quality of care received was the same as for men ^17^, which could also be the case for conditions such as stroke, attention deficit/hyperactivity disorder (ADHD) and arthritis ^1^.

The consequences of the gender health data gap are not limited to clinical settings. Gender bias can impact the quality and representativeness of cause of death statistics in civil registration and vital statistics (CRVS), due to misdiagnosis made by physicians and misreport of certain conditions ^21^. Research into how gender bias in the health system influences the quality of cause of death statistics remains limited ^22^. These biases can also impact population-level summary indicators, which are often used to develop and monitor progress in health, set targets, and develop strategies in national health plans ^23^. Biases can also impact rapidly growing and emerging fields such as digital health, precision medicine, and the use of artificial intelligence (AI), as these technologies still do not account for gender bias detection ^24^.

Some efforts have already been set in motion to start addressing these problems, like the policy of female mice inclusion and sex as a biological variable in clinical trials and scientific studies in 2016 by the U.S. National Institutes of Health (NIH) ^1^ ^,^ ^25^. Group advocates have also focused on raising awareness for specific diseases ^15^ ^,^ ^26^. However, little has been done on the implications of gender health data bias for summary indicators of health. More effort is needed to translate clinical research into population-level statistics ^27^ ^,^ ^28^. In that regard, a closer collaboration between demographers, population health experts and medical specialists is required. For instance, the framework of compression and expansion of morbidity could be used to evaluate whether age at onset and disease progression patterns in men and women are impacted by those data gaps ^29^ ^,^ ^30^. The gender paradox - or the fact that women live longer than men, but in poorer health - could also be tested to understand if it happens partly due to a lack of knowledge or proper understanding of diseases that mainly impact women ^31^ ^,^ ^32^ ^,^ ^33^. Incorporating demographic analysis would allow alternative questions to be asked, such as: are the results upon which research currently builds accurately reflecting the health of populations by age or are they biased due to missing or inconsistent gender evidence? How do those biases, if and when they exist, impact population-level statistics such as healthy life expectancy indicators? If yes, to what extent are policies based on those population-level statistics missing the actual people they target? Lastly, innovative approaches using machine learning and demographic data and technique are a promising field and have already been devised to estimate neonatal mortality, regional disease prevalence estimates, and the role of gender bias on model accuracy ^34^ ^,^ ^35^ ^,^ ^36^ ^,^ ^37^.

The gender health data gap is not trivial and has most likely prevented important advances in knowledge, treatment, and diagnosis of several health conditions. Disciplines, funding agencies, stakeholders, academic institutions, health care systems and the pharmaceutical industry must undertake a concerted effort to identify and bridge the gender health data gap.
